# Advanced muon radiography with compact accelerator system

**Published:** 2004-04-01

**Authors:** Kanetada Nagamine

**Affiliations:** *) Meson Science Laboratory, Institute of Materials Structure Science, KEK, Oho 1-1, Tsukuba, Ibaraki 305-0801, Japan; **) Physics Department, University of California, Riverside, CA 92521, USA

**Keywords:** Muon, radiography, compact accelerator, special nuclear material, security

## Abstract

By using a well-defined mono-energetic, pencil-like, high-energy and intense muon beam, one can realize, via simultaneous measurements of energy-loss and multiple-scattering, a quick and element-selective radiography to detect e.g. a few kg of U which is shielded in a thick Fe container or hidden within 2–3 m of low-Z material. A source of such an ideal beam of muons can be realized in transportable form via truck trailers, by combining a compact 400 MeV electron accelerator for photo ***π***/***μ*** production, a superconducting solenoid for full-solid-angle ***π***/***μ*** capture and transport, a stopping in hot tungsten metal for cooling of energetic ***μ***^+^ to sub-eV ***μ***^+^, and finally a compact linear accelerator for rapid acceleration to 600 MeV. Principle and some details are described.

## Introduction: How muon is powerful to be applied for the thick material radiography

By using elementary particle called muon (***μ***^+^, ***μ***^−^), because of unique mass, electromagnetic-interacting-nature and radioactive-nature, various scientific researches have been realized.1) Among them, a radiography method by using muon to explore inner-structure of a large-scale substance attracts a significant attention.

**Fig. 1:**
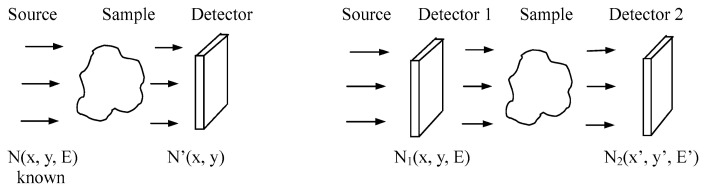
Two types of radiography method; transmission method (left) and perturbation method (right).

There are two types of radiography methods to probe inner-structure of an object, namely a) a transmission method and b) a perturbation method as summarized in Fig. 1.

### a) Transmission method

By using a single detector and a somewhat defined beam of particles or light, the intensity decrease of the beam during a passage through the object is measured in a position-sensitive way. Roentgen photography of human body is a representative example.

### b) Perturbation method

By using two detectors and placing the object in-between, a change of the characteristic of the introduced beam of particles or light is measured during a passage through the object. Typical example of change is as follows: beam divergence by multiple scattering, particle/photon emission, depolarization, etc.

**Fig. 2:**
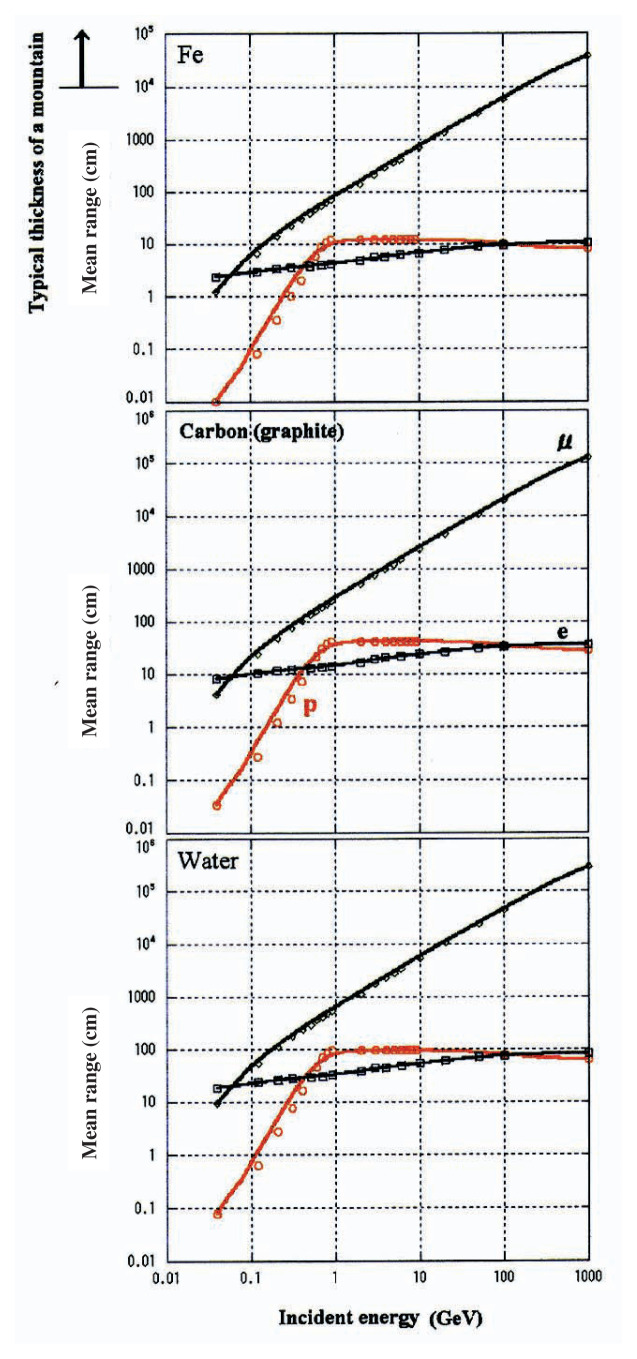
Mean range (cm) of protons(p), electrons (e) and muons (***μ***) through (a) iron, (b) carbon, and (c) water against particle energy (GeV). Here, the mean range is defined as the length of the material where the number of the transmitted particle is attenuated to 50% of the number of the initial injection (taken from reference1)).

In both of these two cases, it is essential for the beam of particles or light to be able to penetrate the object. As summarized in Fig. 2 which represents Range-Energy relation of representative three particles of electron, proton and muon through representative three materials, water, carbon and Fe, muon is the only particle which can penetrate thick materials; at very high energy, electron is converted into photon because of a light-mass-nature and proton is converted into other hadrons because of a strong-interaction-nature.

Thus, excellent features of muon radiography can be summarized as follows: (1) useful for thick materials; any particles other than muon can not be useful for the materials e.g. water (biological substance) with ≥ 1m (E*_μ_* ≥ 0.2 GeV), graphite (volcano, earth crust) with ≥ 30 cm (E*_μ_* ≥ 0.15 GeV), and iron (industrial machinery) with ≥ 10 cm (E*_μ_* ≥ 0.1 GeV); (2) 100% detection efficiency and easiness in track-determination.

By using cosmic-ray muons, pioneering radiography experiments have been carried out to explore inner-structure of large-scale substances; Egyptian Pyramid by Alvarez *et al*.2) and volcanic mountain by Nagamine *et al*.,3) both of which employ a transmission method and laboratory equipment by Borozdin *et al*.4) by employing a perturbation method due to multiple scattering.

## Element-selective radiography with highenergy and high-quality muons

In traversing a thick target, in addition to losing energy, muons are multiply scattered and are deflected by an average angle that increases rapidly with increasing atomic number *Z* of the target. By using a well-defined mono-energetic (energy width of within 100 keV), pencil-like (spot size of smaller than 1 cm^2^), high-energy (600 MeV) and intense (≥ 10^5^/s) muon beam, one can realize a quick and element-selective radiography to detect a few kg of U which is shielded in e.g. a thick Fe container and hidden within 2–3 m of low-Z material.

**Fig. 3:**
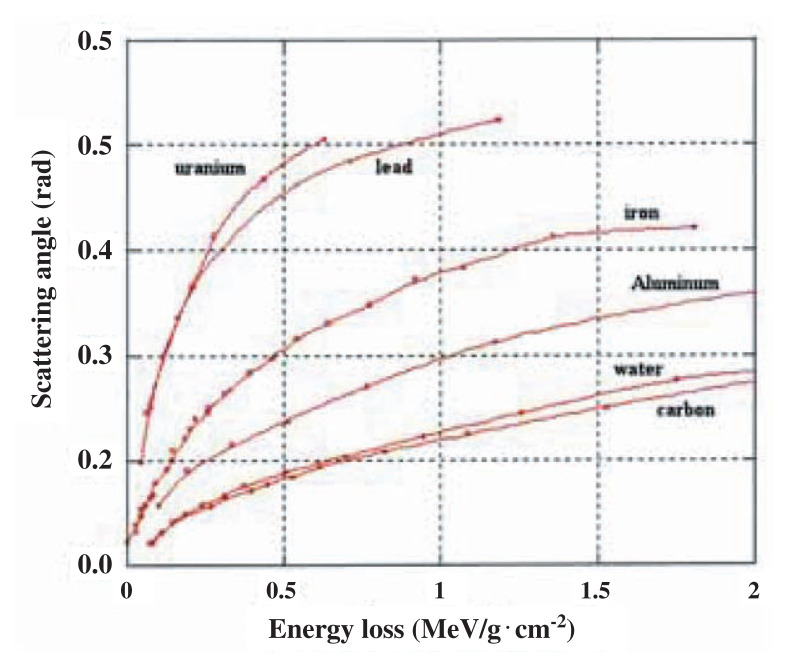
Two-dimensional presentation of energy loss (in terms of differential value) and scattering angle of 100 MeV muons from various materials, where thickness is an implicit parameter.

As summarized in Fig. 3, simultaneous measurements of energy loss and scattering angle can give us a sensitivity to element selection. By obtaining the combined value of (**Δ**E, ***θ***) at each x-y coordinate of the sectional plane of the object one can obtain the value of density length and the information of Z. Thus, element selective radiography can be realized. For instance, a detection of hidden uranium, no matter how thick or thin, can be realized; thin uranium can be discriminated against thick Fe.

## Compact accelerator system for advanced muon radiography

**Fig. 4:**
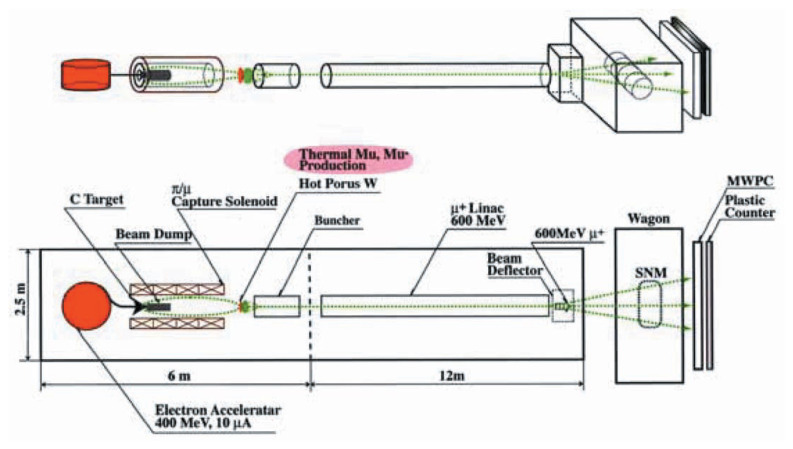
Schematic picture of the proposed system of muon radiography. SNM stands for special nuclear material.

Here, we propose the following scheme of the whole system as schematically described in Fig. 4. Some details of each component are given in the followings.

### a) Compact π/μ generating electron accelerator

Among various possible accelerators that can produce the pions and the muons as their decay-products, we propose a FFAG (fixed-frequency alternating-gradient synchrotron) with a 3T superconducting magnet; a 400 MeV and 10 μA FFAG electron accelerator which, with a cover of radiation shieldings, can be mounted on truck trailer of a 2.5 m × 6m size. Employment of electron accelerator instead of proton is due to a smaller magnetic rigidity.

### b) π/μ capture solenoid

In order to capture produced pions (≥ 10^10^/s in total) with a full-solid-angle acceptance as well as muons produced by the ***π*** → ***μ*** decay in flight, a 3T superconducting solenoid with an aperture of 50 cm will be installed, enclosing a pion-production target and a dump of electron beam. The momentum range upto 200 MeV/c can be captured. By using initial longitudinal momentum, ***π***/***μ*** beam is transported onto the secondary hot W target with the intensity of 10^8^/s.

### c) Muon cooling via stopping in hot tungsten

The primary muon beam (0 ≤ P*_μ_* ≤ 200 MeV/c, **Δ**x and **Δ**y ≅ 2 cm, **Δ*θ***_x_ and **Δ*θ***_y_ ≅ 0.1 rad) transported onto the secondary target can rapidly be cooled by stopping in a stack of hot (2000 K) and thin (50 μm) tungsten foils. There, according to a series of experiments at KEK, ultra-slow ***μ***^+^ is re-emitted in a form of either the near-thermal (0.2 ~ 0.5 eV) Mu (***μ***^+^e^−^) which can be ionized to produce ***μ***^+^ 5) or the Mu^−^ (***μ***^+^e^−^e^−^) with an efficiency of about 5%.6) Thus, “ion source” of the ***μ***^+^ is established with an energy of below 0.5 eV, a beam size of below (1 cm)^2^ and an intensity of 10^6^ ~ 10^7^/s.

### d) μ^+^ acceleration to generate high-quality & high-energy (upto 600 MeV) muons

Once the high-quality ion-source of ***μ***^+^ is prepared as described above, it is possible to connect it to the linear accelerator for muon acceleration. Depending upon acceleration scheme of the connecting linac, it is needed to have a buncher to form muon pulse train in order to be acceptable for the succeeding linac. A high repetition (300 MHz) and high acceleration-gradient (50 MV/m) linac will be employed as the major muon acceleration mechanism.

## Detection system for element-selective radiography

Once muon acceleration is realized following the above-mentioned scenario, high quality and high energy muon beam becomes available with the following advanced feature; a straight pencil-beam character, a small beam-spot (smaller than a few (cm)^2^), small energy width (below a few 100 keV). Then, by placing the object at the beam exit and a single position-sensitive MWPC detector with a plastic-counter complex behind, one can measure an energy loss (E) and a multiple-scattering angle (**Δ*θ***) of the penetrated muons at each muon trajectory, simultaneously.

Actual measurement of both **Δ**E and ***θ*** are as follows:

- **Δ**E; By placing a thin (a few cm thick) plastic counter, one can measure an energy loss in the thin counter of the muon beam penetrated through the object and by using an inverse-proportionality law between **Δ**E and E for the muon with the energy below 200 MeV, one can obtain the energy of the penetrated muon beam. Since the incident energy after the muon accelerator is known (to be 600 MeV), one can obtain energy loss (**Δ**E) of the muon beam through the object. For the total energy of transmitted muon being higher than 200 MeV, iron absorber equivalent to 200 MeV is placed with the same energy-loss counter mentioned above.
- **Δ*θ***; Since incoming beam is known to be like a pencil-beam, the increase of beam size during a passage through the object can provide us a measure of multiple scattering angle.

By the obtained values of (**Δ**E, **Δ*θ***) at each x-y coordinate of the sectional-plane of the object one can obtain the value of density-length and the information of Z as shown in Fig. 3. Thus, element-selective radiography, including the detection of hidden uranium in a thick Fe container, can be realized.

## Conclusion and future perspectives

As shown in Fig. 4, an ideal beam of muons can be realized in transportable form via truck trailers, by combining presently available technologies including a compact 400 MeV electron accelerator, ***π***/***μ*** production by a photo-nuclear reaction, full-solid-angle ***π***/***μ*** capture and transport by means of a superconducting solenoid, cooling of energetic ***μ***^+^ to sub-eV ***μ***^+^ in the form of ***μ***^+^ or Mu^−^(***μ***^+^e^−^e^−^) by stopping in hot tungsten metal, and finally rapid acceleration to 600 MeV by a compact linear accelerator.

The muon beam can penetrate low Z-materials like water, oil, coal or sand fully occupying a thickness of a few m and iron of 50 cm. A magnetic beam deflector is used to scan e.g. a 2.5 × 3.0 × 12.0 m cargo container in 20 seconds. The energy losses and multiple-scattering angles of the transmitted muons are detected by a plastic counter system and a multiwire proportional counter (MWPC) to determine the average atomic number and thickness of the material. The known stability of operation of the accelerator systems and scanning-detector system makes it possible to operate almost automatically.

This method is also applicable to solve the problems in industrial machineries, thus contributing a wealth of human life: a) some fault in the part of nuclear reactor can be found; b) static as well as dynamic inside-behaviour of iron blast furnace can be probed to provide an information of the safe and efficient iron production; c) by increasing muon energy furthermore, as predicted in the earlier publication,1) one can investigate, more precisely and more efficiently compared to the case of cosmic-ray muon use, insider nature of volcano for eruption prediction.
